# Association between plasma Netrin‐1 levels and motor and nonmotor symptoms in Parkinson's disease

**DOI:** 10.1111/cns.70022

**Published:** 2024-08-30

**Authors:** Ye Hua, Min Wang, Qingyu Yao, Bin Hu, Feng Lu, Yi Fan, Weifeng Lu

**Affiliations:** ^1^ Department of Neurology The Second Affiliated Hospital of Soochow University Suzhou China; ^2^ Department of Neurology Wuxi No. 2 People's Hospital, Jiangnan University Medical Center Wuxi China; ^3^ Department of Pharmacology Neuroprotective Drug Discovery Center of Nanjing Medical University, Nanjing Medical University Nanjing China

**Keywords:** Netrin‐1, neurodegeneration, Parkinson's disease, UPDRS

## Abstract

**Background:**

Parkinson's disease (PD) is a prevalent neurodegenerative disorder characterized by dopaminergic neuron degeneration and diverse motor and nonmotor symptoms. Early diagnosis and intervention are crucial but challenging due to reliance on clinical presentation. Recent research suggests potential biomarkers for early detection, including plasma netrin‐1 (NTN‐1), a protein implicated in neuronal survival.

**Methods:**

This cross‐sectional study recruited 105 PD patients and 65 healthy controls, assessing plasma NTN‐1 levels and correlating them with clinical characteristics. Statistical analyses explored associations between NTN‐1 levels and PD symptoms, considering demographic factors.

**Results:**

PD patients exhibited significantly lower plasma NTN‐1 levels compared to controls. NTN‐1 demonstrated moderate potential as a PD biomarker. Positive correlations were found between NTN‐1 levels and motor, depression, and cognitive symptoms. Multiple regression analysis revealed disease duration and NTN‐1 levels as key factors influencing symptom severity. Gender also impacted symptom scores.

**Conclusion:**

Reduced plasma NTN‐1 levels correlate with PD severity, suggesting its potential as a biomarker. However, further research is needed to elucidate the roles of NTN‐1 in PD pathophysiology and validate its diagnostic and therapeutic implications. Understanding the involvement of NTN‐1 may lead to personalized management strategies for PD.

## Graphical abstract

Parkinson's disease (PD) is characterized by neuron degeneration and diverse symptoms, posing diagnostic challenges. Plasma netrin‐1 (NTN‐1) emerges as a potential biomarker for early detection. A study with 105 PD patients and 65 controls shows lower NTN‐1 levels in PD, correlating with symptom severity and demographic factors.

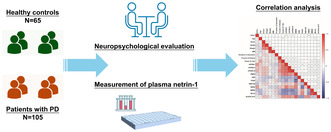

## 1 INTRODUCTION

Parkinson's disease (PD), recognized as the second most common neurodegenerative disease after Alzheimer's disease, affects approximately 1% of the population over 60.^1^ Pathologically, PD is characterized by the progressive degeneration of dopaminergic neurons in the substantia nigra, and the accumulation of intraneuronal α‐synuclein inclusions known as Lewy bodies.^2^, ^3^ Clinically, PD primarily presents with motor symptoms such as bradykinesia, resting tremor, muscle rigidity, and postural instability. However, growing evidence underscores the significance of nonmotor symptoms, such as olfactory dysfunction, sleep disturbances, constipation, depression, and dysautonomia, which arise from α‐synuclein pathology affecting additional brain regions.^4^ This heightened recognition of nonmotor symptoms emphasizes the complexity of PD and the need for comprehensive treatment strategies.

Early diagnosis and intervention are critical in PD to optimize patient outcomes. However, current diagnostic methods rely largely on clinical presentation, which emerges after subsequent dopaminergic neuron loss. Recent advancements in research offer promising avenues for identifying biomarkers for early detection. Advanced neuroimaging techniques, such as [18F] DOPA PET for assessing dopaminergic loss and MRI for detecting early brain changes, hold potential in biomarker discovery efforts.^5^ Additionally, investigations into biochemical markers in plasma, a minimally invasive alternative to cerebrospinal fluid (CSF), show promise due to its accessibility and cost‐effectiveness. Candidates such as α‐synuclein, neurofilament light chain (NfL), and dopamine metabolites are being explored for their diagnostic utility in PD.^6^ Research into inflammatory markers^7^ and analyses of the gut microbiome^8^ is also ongoing. However, plasma and other biomarker currently serve as adjuncts to clinical evaluation rather than stand‐alone diagnostic tools.

Interestingly, studies have uncovered a notable phenomenon in PD: a reduction in netrin‐1 (NTN‐1) levels, specifically in the substantia nigra, where dopamine neurons undergo degeneration.^9^, ^10^, ^11^ Conversely, animal models have shown that elevating NTN‐1 levels can protect against the degeneration of dopamine neurons, indicating NTN‐1's potential as a therapeutic target for enhancing neuron survival in PD. Our prior research has also expanded upon this link, demonstrating that decreased plasma NTN‐1 levels correlate with early dopaminergic neuron injury in PD.^12^ Intriguingly, we observed a negative correlation: higher plasma NTN‐1 levels seemed associated with more severe motor symptoms in PD patients.

NTN‐1 is a multifunctional protein crucial in both developmental processes and adult physiology.^13^ As a member of the netrin family, NTN‐1 shares structural similarities with laminin‐related secreted proteins and contains laminin‐like domains essential for binding to receptors and initiating signaling cascades. During neural development, NTN‐1 guides axons through interactions primarily with DCC and UNC5 family receptors, influencing cell survival, growth cone dynamics, and cytoskeletal organization via pathways, such as PI3K/Akt and MAPK/ERK.^14^ Beyond developmental roles, NTN‐1 promotes angiogenesis by stimulating endothelial cell migration and vessel formation, interacting with integrins and other receptors.^15^ Consequently, the presence of NTN‐1 in plasma intimates broader systemic roles, potentially influencing inflammatory responses^16^ and tissue‐repair processes,^17^ while serving as a biomarker indicative of pathological conditions.^18^, ^19^, ^20^, ^21^

Particularly, the pervasive expression of NTN‐1 throughout the nervous system and beyond, engenders significant impact on various facets of PD.^13^ Within the central nervous system, NTN‐1 assumes a regulatory mantle, shaping neuronal connectivity within motor circuits and potentially influencing motoric symptoms.^22^ Furthermore, NTN‐1 expression in cortical and limbic regions may contribute to nonmotor symptoms such as cognitive decline and mood disturbances.^22^ Peripherally, NTN‐1 signaling in nerves, muscles, and the gastrointestinal tract may elucidate diverse PD manifestations, including gastrointestinal malfunctions.^11^ Thus, our ongoing research endeavors to elucidate the correlation between NTN‐1 levels and both motor and nonmotor symptoms in PD. Through expanding our investigative purview and synthesizing observations of nonmotor symptomatology, we aim to attain a comprehensive comprehension of NTN‐1's integral role in the disease's progression.

## 2 METHODS

### 2.1 Standard protocol approvals, registrations, and patient consent

Participants were consecutively recruited from the Second People's Hospital of Wuxi for this study. The study protocol obtained approval from the Institutional Ethics Board Committee of the Second People's Hospital of Wuxi (approval document number 2020‐Y‐2). Prior to enrollment, all participants provided written informed consent.

### 2.2 Study population

**FIGURE 1:**
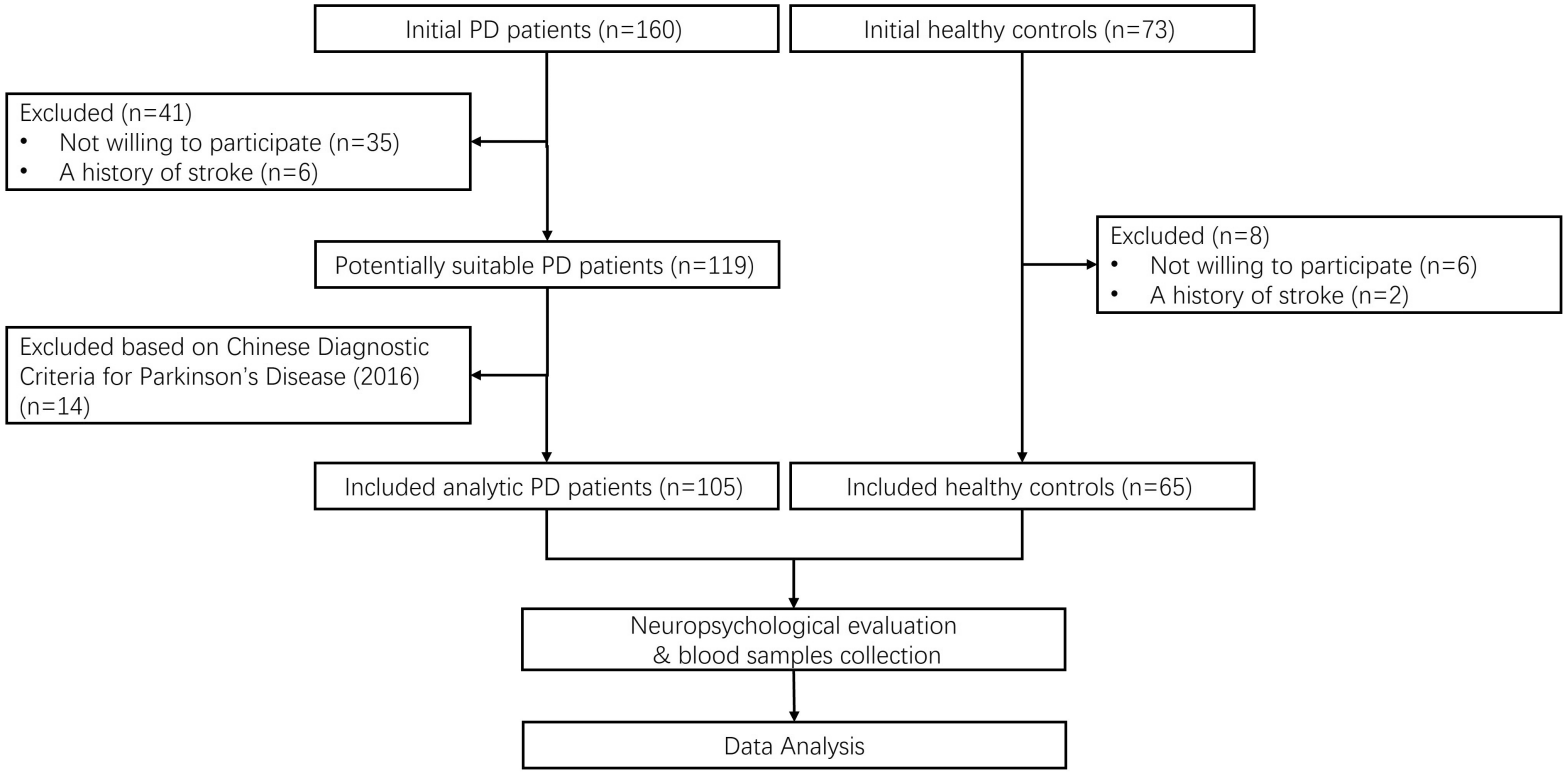
Participant flowchart. PD, Parkinson's disease.

See Figure 1 for a flowchart outlining the selection process of this study. All participants were recruited from the Second People's Hospital of Wuxi between October 2020 and September 2023. Patients diagnosed with PD were classified according to Chinese Diagnostic Criteria for Parkinson's Disease (2016), which utilized the clinical diagnostic criteria for Parkinson's disease of UK Parkinson's Disease Society Brain Bank and Movement Disorder Society (2015) as references. We carefully selected a control group of healthy individuals from the hospital's medical checkup center, ensuring age, gender, and education level matched with the PD group. Prior to enrollment, all participants underwent a thorough medical history review to verify eligibility. Exclusion criteria for both groups encompassed familial PD, other Parkinsonian syndromes, secondary Parkinsonism due to diverse etiologies, severe chronic illnesses, and a history of stroke, tumors, or mental health disorders.

### 2.3 Neuropsychological evaluation

Each patient underwent a thorough neurological assessment conducted by a specialist in movement disorders. We evaluated disease severity using the Hoehn‐Yahr (H‐Y) stages. Motor symptoms were assessed using the Unified Parkinson's Disease Rating Scale (UPDRS), while nonmotor symptoms were evaluated using the Non‐Motor Symptoms Scale (NMSS). Additionally, we utilized specific assessments to explore various aspects of PD: the Hyposmia Rating Scale (HRS) for olfactory function, the Parkinson's Disease Sleep Scale (PDSS) to evaluate sleep quality, the Rapid Eye Movement Sleep Behavior Disorder Screening Questionnaire (RBD‐SQ) to identify potential REM sleep abnormalities, the Hamilton Depression Scale (HAMD) for depressive symptoms, the Mini‐Mental State Examination (MMSE) and the Montreal Cognitive Assessment (MoCA) for cognitive function, and the Scales for Outcomes in Parkinson's disease‐Autonomic (SCOPA‐AUT) scale to assess autonomic symptoms. Notably, all PD patients were evaluated during their “off” medication phase.

### 2.4 Measurement of plasma NTN‐1

Three milliliters of whole blood were meticulously collected from each participant using EDTA‐containing vacutainer tubes (Labtub, Shanghai, China). Following a 1‐hour resting period at room temperature, the samples underwent centrifugation at 3000 rpm for 15 min. The resulting separated plasma was then carefully aliquoted and stored at −80°C. Subsequently, we quantified NTN‐1 levels using a commercially available enzyme‐linked immunosorbent assay (ELISA) kit (CSB‐E11899h, Cusabio Biotech, Wuhan, China).

### 2.5 Statistical analysis

The statistical analyses were performed using GraphPad Prism 9.3. The Kolmogorov–Smirnov test was used to evaluate the distribution of variables. Those variables exhibiting a normal distribution were described as mean ± SEM and subjected to comparison via two‐tailed *t*‐tests. Conversely, variables not adhering to a normal distribution were described as the median and interquartile range (IQR, delineating 25th and 75th percentiles) and analyzed utilizing Mann–Whitney *U* tests. Categorical data were analyzed using chi‐squared tests.

To ascertain the diagnostic efficacy of NTN‐1 for PD, receiver operating characteristic (ROC) curves were constructed. Associations between plasma NTN‐1 levels and clinical scores were examined using Spearman's rank correlation coefficients. Furthermore, a multiple linear regression analysis was undertaken to explore the influence of age, gender, body mass index (BMI), disease duration, and NTN‐1 levels on clinical scores. A partial correlation analysis was employed to discern the specific association between NTN‐1 and clinical scores, adjusting for potential confounders such as disease duration and age. The threshold for statistical significance was established at *p* < 0.05.

## 3 RESULTS

### 3.1 Clinical characteristics of the study population

We recruited a total of 170 participants, including 65 healthy controls (HC) and 105 patients diagnosed with PD. Table 1 summarizes their demographic and clinical characteristics. Although the PD group had a higher male‐to‐female ratio than the HC group (*χ* ^*2*^ = 1.322, *p* = 0.250; chi‐squared tests), this difference did not reach statistical significance. Similarly, there were no statistically significant differences in age (*t* = 1.305, *p* = 0.194; two‐tailed *t*‐tests), height (*t* = 0.429, *p* = 0.669; two‐tailed *t*‐tests), weight (*t* = 0.077, *p* = 0.939; two‐tailed *t*‐tests), or BMI (*t* = 0.534, *p* = 0.594; two‐tailed *t*‐tests) between the two groups. Notably, the PD group exhibited slightly lower education levels, although this difference did not achieve statistical significance (*U* = 3359, *p* = 0.860; Mann–Whitney *U* tests).

**TABLE 1:** Demographic and clinical features of the PD patients.

	HC patients	PD patients	*p*
Numbers	65	105	
Age (years)	68.5 ± 8.4	70.3 ± 8.9	0.194
Gender (male/female)	37/28	69/36	0.250
Height (cm)	167.6 ± 0.9	167.1 ± 0.7	0.669
Weight (kg)	64.78 ± 1.16	64.90 ± 0.97	0.939
BMI	22.97 ± 2.24	23.21 ± 2.99	0.594
Duration of education (years)	12.0 (7.5–12.0)	9.0 (9.0–12.0)	0.860
Disease duration (years)	–	2.5 (1.0–5.0)	
H‐Y stages	–	2.0 (1.5–2.5)	
UPDRSI score	–	2.0 (1.0–3.0)	
UPDRSII score	–	8.0 (5.0–12.5)	
UPDRSIII score	–	17.0 (11.0–24.5)	
NMSS score^a^	–	42.0 (28.0–58.0)	
HRS score^a^		19.0 (12.0–24.0)	
PDSS score^a^	–	124.0 (113.0–132.0)	
RBD‐SQ score^a^	–	2.0 (1.0–6.0)	
HAMD score^a^	–	3.0 (2.0–7.0)	
MMSE score^a^	–	28.0 (25.0–29.0)	
MoCA score^a^	–	23.0 (20.0–26.0)	
SCOPA‐AUT score^a^	–	31.0 (27.0–36.0)	

Abbreviations: BMI, body mass index; HAMD, Hamilton Depression Scale; HC, healthy controls; HRS, Hyposmia Rating Scale; H‐Y, Hoehn‐Yahr; MMSE, Mini‐Mental State Examination; MoCA, Montreal Cognitive Assessment; NMSS, Non‐Motor Symptoms Scale; PD, Parkinson's disease; PDSS, Parkinson's Disease Sleep Scale; RBD‐SQ, Rapid Eye Movement Sleep Behavior Disorder Screening Questionnaire; SCOPA‐AUT, Scales for Outcomes in Parkinson's disease – Autonomic; UPDRS, Unified Parkinson's Disease Rating Scale.

^a^ Two patients with H‐Y stage 5 were unable to speak and were not included in the analysis.

### 3.2 NTN‐1 decrease in the plasma of PD patients

**FIGURE 2:**
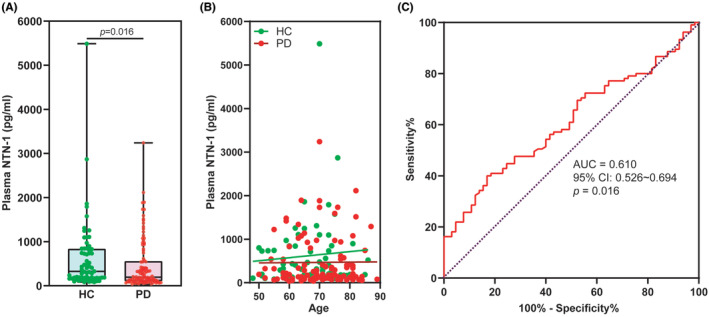
Plasma NTN‐1 levels and ROC curvesdistinguishing PD patients from HC. (A) Scatter diagram of plasma NTN‐1 levels between the HC and PD groups. Data are presented as dot plots in each group and analyzed by Mann–Whitney *U* test. The whiskers in the box plots indicate the values from minimum to maximum, and the center line indicates the median. (B) Distribution of plasma NTN‐1 levels by age. CROC curves of plasma NTN‐1 levels for distinguishing PD patients from HC. AUC, area under the ROC curve; HC, healthy control; PD, Parkinson's disease; NTN‐1, netrin‐1.

The plasma levels of NTN‐1 were significantly lower in patients with PD compared to HC (Figure 2A). Specifically, the median concentration of NTN‐1 in the PD group was 194.0 pg/mL (with a range of 105.4–558.6 pg/mL), while the HC group had a median of 325.7 pg/mL (with a range of 176.1–836.0 pg/mL) (*p* = 0.016, Mann–Whitney *U* test). Notably, linear correlation analysis revealed that plasma NTN‐1 levels were independent of age in both HC and PD groups (Figure 2B). Furthermore, our analysis using receiver operating characteristic (ROC) curves demonstrated that the AUC to discriminating PD from HC participants was 0.610 (95% CI: 0.526–0.694, *p* = 0.016) for plasma NTN‐1 (Figure 2C). By using a cutoff value of 157.0 pg/mL, plasma NTN‐1 exhibited a sensitivity of 40.0% (CI: 31.0%–49.6%) and a specificity of 83.1% (CI: 72.2%–90.3%) for PD diagnosis. These findings support our previous work^12^ and suggest that plasma NTN‐1 levels may hold promise as a biomarker for PD.

### 3.3 Association of plasma NTN‐1 with clinical characteristics in PD patients

**FIGURE 3:**
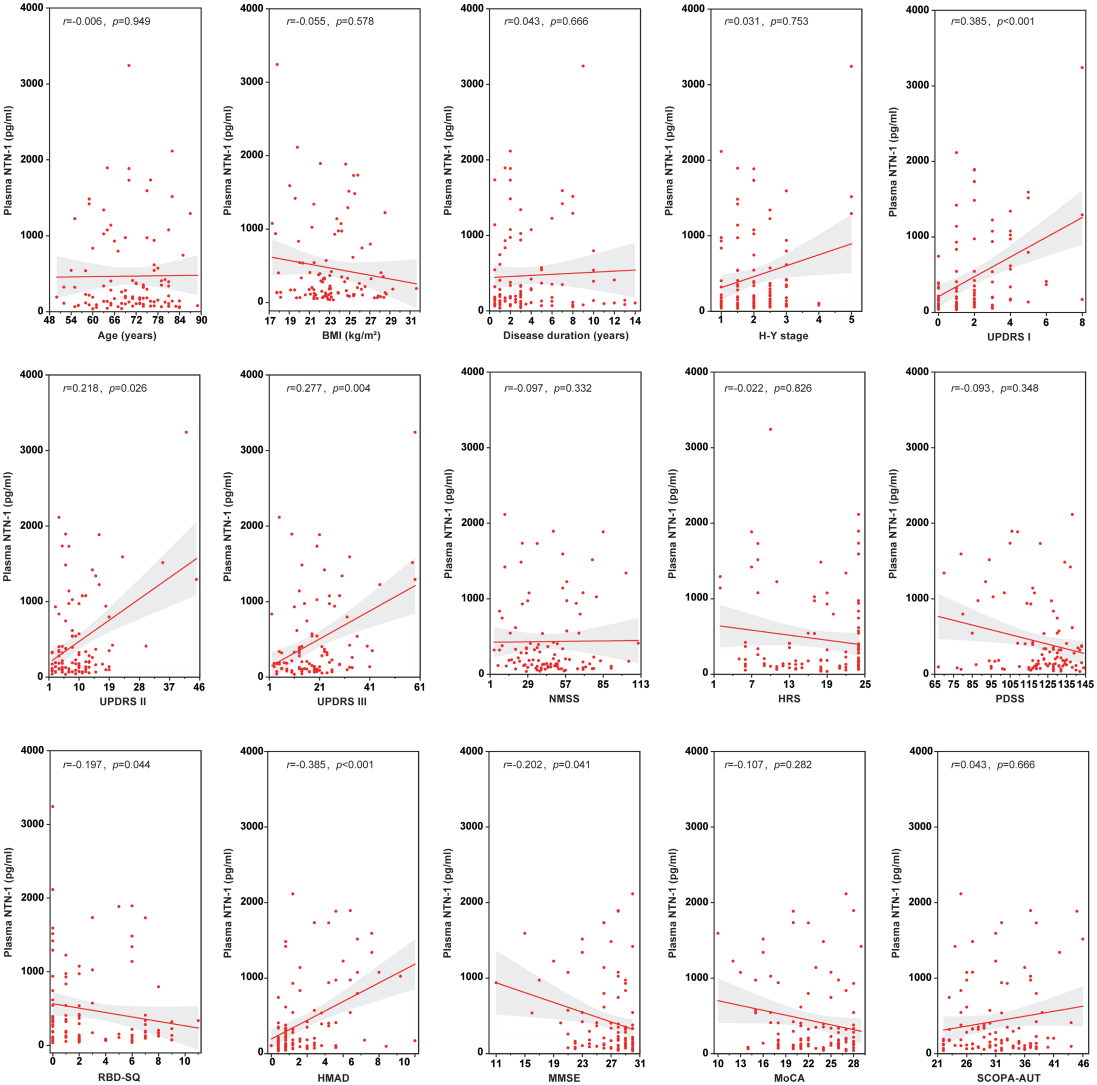
Scattering plot of plasma NTN‐1 levels provided by clinical characteristics in PD patients. Linear regression lines with 95% confidence intervals are fitted (a gray area). Spearman rank correlation coefficient (*r*) and corresponding *p*‐value are reported.

The correlations between plasma NTN‐1 levels and various clinical features in PD patients are summarized in Figure 3. Spearman correlation coefficients revealed significant positive correlations between NTN‐1 and scores on the UPDRS I (movement complexity; *r* = 0.385, *p* < 0.001), UPDRS II (activities of daily living; *r* = 0.218, *p* = 0.026), and UPDRS III (mental impairment; *r* = 0.277, *p* = 0.004), indicating that higher NTN‐1 levels are associated with more severe motor and mental symptoms. Additionally, a positive correlation was also found with HAMD scores (*r* = 0.385, *p* < 0.001), suggesting a link with depression symptoms. Conversely, NTN‐1 levels showed negative correlations with RBD‐SQ (screening for REM sleep behavior problems; *r* = −0.197, *p* = 0.044) and MMSE (cognitive function; *r* = −0.202, *p* = 0.041) scores, implying that higher NTN‐1 levels might be associated with worse performance in these areas. Notably, there were no significant correlations between plasma NTN‐1 and age, BMI, disease duration, H‐Y stage (disease severity), non‐motor symptoms score (NMSS), hyposmia rating scale (HRS), sleep quality scale (PDSS), MoCA (cognitive function), or autonomic symptoms score (SCOPA‐AUT).

### 3.4 Correlation of plasma NTN‐1 and other demographic characteristics with symptom scores

**FIGURE 4:**
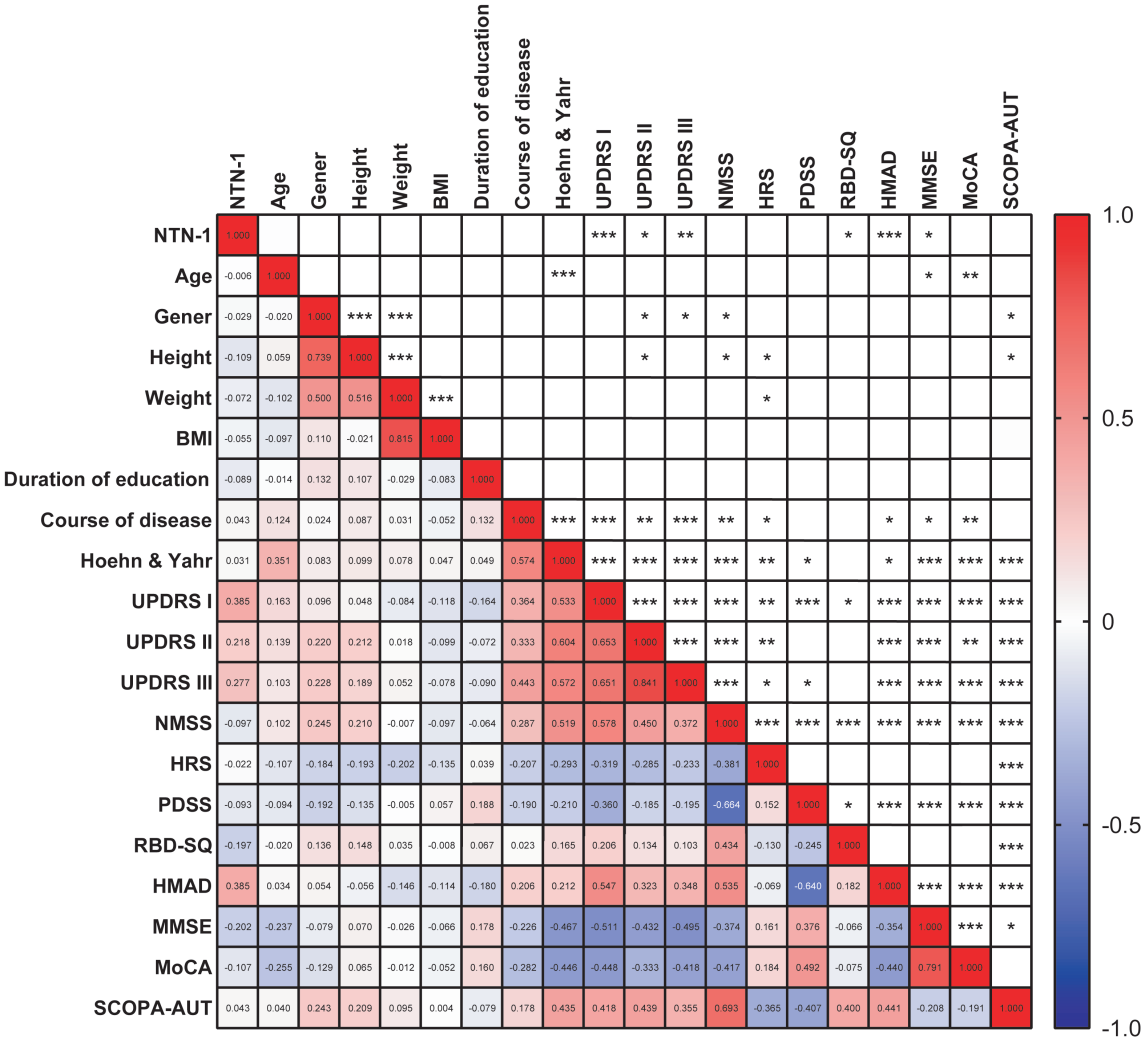
Spearman correlation matrix of clinical characteristics in PD patients. The lower‐left numbers in this matrix are Spearman rank correlation coefficient (*r*) and the upper‐right asterisk are corresponds to the corresponding *p*‐value. ***p < 0.001, **p < 0.01, *p < 0.05.

Through a linear correlation matrix, we analyzed all observed clinical characteristics, including NTN‐1. We discovered that these symptom scores were also influenced by age, gender, height, weight, and disease duration (Figure 4). To address potential confounding factors and precisely examine the association between NTN‐1 levels and motor and nonmotor symptoms, we conducted multiple linear regression analysis. In this analysis, we considered various symptom scores as dependent variables, while age, gender, BMI, disease duration, and NTN‐1 level served as independent variables.

Multiple linear regression analysis revealed interesting associations between factors and PD symptoms (except RBD‐SQ) (see Table 2). PlasmNTN‐1 levels affected motor function (H‐Y stages and UPDRS), depression (HAMD), and cognition function (PDSS, MMSE, and MoCA). Disease duration worsened most symptoms, while age only affected the H‐Y stages. Gender impacted activities of daily living, mental impairment, nonmotor symptoms, and autonomic symptoms (UPDRS II, III, NMSS, and SCOPA‐AUT). BMI had no significant effect on any PD symptom scores.

**TABLE 2:** Multiple linear regression analysis for the effects of independent variables on symptom scores of PD patients.

	Independent variable	*B*	*β*	*t*	*p*	*F*	Adj *R* ^2^
H‐Y stages	NTN‐1	0.000	0.214	2.866	0.005	16.770***	0.431
	Age	0.029	0.300	3.993	<0.001		
	Disease duration	0.135	0.525	7.007	<0.001		
	Gender	0.150	0.082	1.108	0.271		
	BMI	0.039	0.136	1.792	0.076		
UPDRS I score	NTN‐1	0.001	0.383	4.533	<0.001	8.665***	0.269
	Age	0.024	0.120	1.408	0.162		
	Disease duration	0.157	0.294	3.458	<0.001		
	Gender	0.414	0.110	1.302	0.196		
	BMI	−0.067	−0.111	−1.296	0.198		
UPDRS II score	NTN‐1	0.005	0.394	4.753	<0.001	9.879***	0.299
	Age	0.115	0.137	1.640	0.104		
	Disease duration	0.727	0.324	3.896	<0.001		
	Gender	2.625	0.166	2.009	0.047		
	BMI	−0.134	−0.053	−0.632	0.529		
UPDRS III score	NTN‐1	0.007	0.365	4.443	<0.001	10.359***	0.310
	Age	0.104	0.079	0.960	0.339		
	Disease duration	1.379	0.395	4.785	<0.001		
	Gender	4.640	0.188	2.300	0.024		
	BMI	−0.052	−0.013	−0.158	0.874		
NMSS score	NTN‐1	0.001	0.012	0.130	0.897	4.698***	0.153
	Age	0.173	0.066	0.714	0.477		
	Disease duration	2.277	0.326	3.538	<0.001		
	Gender	13.215	0.270	2.955	0.004		
	BMI	−0.870	−0.111	−1.201	0.233		
HRS score	NTN‐1	−0.002	−0.140	−1.471	0.144	2.731*	0.077
	Age	−0.118	−0.157	−1.644	0.103		
	Disease duration	−0.372	−0.187	−1.958	0.053		
	Gender	−2.335	−0.166	−1.757	0.082		
	BMI	−0.300	−0.134	−1.393	0.167		
PDSS score	NTN‐1	−0.008	−0.237	−2.495	0.014	3.020*	0.090
	Age	−0.068	−0.034	−0.353	0.725		
	Disease duration	−1.414	−0.262	−2.746	0.007		
	Gender	−4.513	−0.120	−1.262	0.210		
	BMI	0.051	0.008	0.088	0.930		
RBD‐SQ score	NTN‐1	−0.001	−0.168	−1.698	0.093	1.011	0.001
	Age	0.017	0.048	0.486	0.628		
	Disease duration	0.037	0.040	0.406	0.686		
	Gender	0.850	0.130	1.319	0.190		
	BMI	−0.039	−0.037	−0.371	0.712		
HMAD score	NTN‐1	0.004	0.424	4.824	<0.001	6.787***	0.221
	Age	−0.019	−0.040	−0.453	0.652		
	Disease duration	0.302	0.235	2.656	0.009		
	Gender	0.750	0.083	0.950	0.344		
	BMI	−0.201	−0.140	−1.573	0.119		
MMSE score	NTN‐1	−0.002	−0.252	−2.639	0.010	2.824*	0.082
	Age	−0.059	−0.143	−1.483	0.141		
	Disease duration	−0.232	−0.211	−2.203	0.030		
	Gender	−0.190	−0.025	−0.259	0.796		
	BMI	−0.026	−0.021	−0.216	0.829		
MoCA score	NTN‐1	−0.002	−0.208	−2.230	0.028	3.863**	0.123
	Age	−0.089	−0.179	−1.900	0.060		
	Disease duration	−0.365	−0.276	−2.938	0.004		
	Gender	−1.078	−0.116	−1.249	0.215		
	BMI	−0.129	−0.087	−0.926	0.357		
SCOPA‐AUT score	NTN‐1	0.002	0.159	1.684	0.095	3.346**	0.103
	Age	0.030	0.045	0.474	0.637		
	Disease duration	0.443	0.251	2.645	0.010		
	Gender	3.079	0.249	2.646	0.009		
	BMI	−0.001	0.000	−0.003	0.998		

Abbreviations: BMI, body mass index; HAMD, Hamilton Depression Scale; HRS, Hyposmia Rating Scale; H‐Y, Hoehn‐Yahr; MMSE, Mini‐Mental State Examination; MoCA, Montreal Cognitive Assessment; NMSS, Non‐Motor Symptoms Scale; NTN‐1, netein‐1; PD, Parkinson's disease; PDSS, Parkinson's Disease Sleep Scale; RBD‐SQ, Rapid Eye Movement Sleep Behavior Disorder Screening Questionnaire; SCOPA‐AUT, Scales for Outcomes in Parkinson's disease – Autonomic; UPDRS, Unified Parkinson's Disease Rating Scale.

* *p* < 0.05;

** *p* < 0.01;

*** *p* < 0.001.

### 3.5 Correlation between plasma NTN‐1 with motor and nonmotor features in PD patients

We conducted partial correlation analysis, considering disease duration and its interactions with age. This analysis revealed a correlation between NTN‐1 levels and PD symptoms (see Table 3). Specifically, elevated NTN‐1 levels were associated with increased severity in motor function (H‐Y stage and UPDRS scores) and potential depression (HAMD score). Conversely, they were also linked to lower scores on PDSS, MMSE, and MoCA, suggesting poorer cognitive function. Notably, we did not observe any significant effect on sleep disorders or autonomic neurological symptoms.

**TABLE 3:** Spearman and partial correlation analyses between NTN‐1 levels and symptom scores of PD patients.

	Spearman correlation		Partial correlation (adjusted for disease duration)		Partial correlation (adjusted for disease duration and age)	
	*r*	*P*	*r*	*P*	*r*	*P*
H‐Y stages	0.031	0.753	**0.237**	**0.016**	**0.251**	**0.011**
UPDRSI score	**0.385**	**<0.001**	**0.419**	**<0.001**	**0.423**	**<0.001**
UPDRSII score	**0.218**	**0.026**	**0.426**	**<0.001**	**0.430**	**<0.001**
UPDRSIII score	**0.277**	**0.004**	**0.399**	**<0.001**	**0.400**	**<0.001**
NMSS score	−0.097	0.332	0.031	0.758	0.032	0.751
HRS score	−0.022	0.826	−0.123	0.214	−0.124	0.214
PDSS score	−0.093	0.348	**−0.249**	**0.012**	**−0.250**	**0.012**
RBD‐SQ score	**−0.197**	**0.044**	−0.165	0.094	−0.166	0.095
HAMD score	**0.385**	**<0.001**	**0.446**	**<0.001**	**0.446**	**<0.001**
MMSE score	**−0.202**	**0.041**	**−0.255**	**0.010**	**−0.259**	**0.009**
MoCA score	−0.107	0.282	**−0.212**	**0.032**	**−0.217**	**0.029**
SCOPA‐AUT score	0.043	0.666	0.173	0.083	0.173	0.083

Abbreviations: BMI, body mass index; HAMD, Hamilton Depression Scale; HRS, Hyposmia Rating Scale; H‐Y, Hoehn‐Yahr; MMSE, Mini‐Mental State Examination; MoCA, Montreal Cognitive Assessment; NMSS, Non‐Motor Symptoms Scale; NTN‐1, netein‐1; PD, Parkinson's disease; PDSS, Parkinson's Disease Sleep Scale; RBD‐SQ, Rapid Eye Movement Sleep Behavior Disorder Screening Questionnaire; SCOPA‐AUT, Scales for Outcomes in Parkinson's disease – Autonomic; UPDRS, Unified Parkinson's Disease Rating Scale.

The bolded data indicates a significant difference.

## 4 DISCUSSION

Our investigation confirms a marked decrease in plasma NTN‐1 levels among PD patients compared to HC, with no notable differences in demographic characteristics between the groups. This decline in NTN‐1 levels highlights its potential as a biomarker for PD. This assertion is supported by the moderate discriminatory power observed in the AUC analysis, along with promising diagnostic implications evidenced by sensitivity and specificity values at a cutoff threshold of 157.0 pg/mL.

The survival of dopaminergic neurons heavily depends on NTN‐1 signaling through its DCC receptor. Previous research emphasizes the survival benefits conferred by robust NTN‐1/DCC signaling pathways in maintaining dopaminergic neuron integrity. A deficiency in NTN‐1 correlates with a significant loss of dopaminergic neurons in the midbrain region.^9^ Conversely, NTN‐1 overexpression has been shown to protect these neurons in animal models of PD.^10^ Our earlier research has also identified a noticeable imbalance between Netrin‐1 and its receptor DCC within damaged dopaminergic brain regions, suggesting this imbalance is a characteristic feature of PD pathology.^12^ Despite extensive research into the neuroprotective functions of NTN‐1 in the brain, the relationship between plasma NTN‐1 levels and the progression of PD remains unclear.

In our study of PD patients, Spearman's correlation analysis revealed significant positive associations between NTN‐1 levels and UPDRS and HAMD scores. Additionally, significant negative associations were observed with RBD‐SQ and MMSE scores. Interestingly, no significant correlation was found with the commonly used H‐Y stages for assessing motor symptom severity. Multiple regression analysis identified disease duration and NTN‐1 levels as key factors influencing symptom severity, with gender, age, and BMI also playing roles. Subsequent partial correlation analysis revealed a positive correlation between higher NTN‐1 levels and increased severity of motor symptoms (H‐Y stages and UPDRS scores), depression (HAMD scores), and cognitive function (MMSE and MoCA scores) in PD patients. These findings suggest NTN‐1 plays a multifaceted role in PD, impacting motor, emotional, and cognitive functions.

Given the high expression of NTN‐1 on nigrostriatal dopaminergic neurons,^10^ its association with motor dysfunction in PD is unsurprising. Furthermore, our study suggests that plasma NTN‐1 levels primarily relate to nonmotor symptoms, particularly emotional and cognitive function. Recent research also indicates an involvement of NTN‐1 in emotional^22^, ^23^ and cognitive functions.^18^, ^19^ Interestingly, decreased NTN‐1 levels correlate with cognitive decline in Alzheimer's disease and mild cognitive impairment. Conversely, NTN‐1 may confer neuroprotective effects, potentially explaining its association with enhanced cognitive function.

We observed a correlation between plasma NTN‐1 levels and sleep‐related scores, particularly PDSS scores. Currently, there is no direct evidence in the literature regarding the correlation between NTN‐1 and sleep regulation. However, NTN‐1 is predominantly recognized for guiding axon growth,^24^, ^25^ regulating inflammation,^16^ and exerting neuroprotective effects.^26^ Sleep regulation entails intricate mechanisms, and while NTN‐1 may indirectly impact neuronal function, further research is needed to clarify its precise role in sleep regulation.

NTN‐1 is not limited to the central nervous system; it is also prominently expressed in Schwann cells of the peripheral nervous system.^21^, ^27^ It exhibits multifaceted actions, including facilitating nerve innervation, promoting angiogenesis, and enhancing the stabilization of nerve barriers following injury. Previous studies suggest a potential link between reduced NTN‐1 levels and the severity of chronic constipation in PD patients.^11^ Nevertheless, our study did not find a significant association between NTN‐1 levels and autonomic function scores (specifically NMSS and SCOPA‐AUT scores). Notably, the evaluation of autonomic symptoms relied heavily on subjective rating scales in this study, lacking comprehensive support from imaging, functional assessments, or pathology. Future investigations using rigorous methodologies are crucial to unravel the complex relationship between plasma NTN‐1 levels and peripheral nonmotor symptoms, particularly chronic constipation.

Despite being traditionally associated with neuroprotection and neuronal survival,^16^, ^17^, ^26^ increased NTN‐1 signaling is indeed linked to motor or nonmotor neurological symptoms in PD. Several neuroprotective factors, including brain‐derived neurotrophic factor (BDNF),^28^ glial cell‐derived neurotrophic factor (GDNF),^29^ vascular endothelial growth factor (VEGF),^30^, ^31^ and cystatin C (Cys C),^32^, ^33^ increase in plasma levels with symptom severity, likely as compensatory responses to neurodegeneration.^34^ As the disease progresses, these factors play crucial roles in supporting neuronal health and reducing neuroinflammation. Elevated NTN‐1 levels may also reflect compensatory mechanisms in response to ongoing neurodegeneration, contributing to motor impairments in PD.^12^ Further research is needed to understand the relationship between plasma NTN‐1 levels and their implications for PD diagnosis and treatment.

Despite the compelling findings, several study limitations should be acknowledged. The cross‐sectional design precludes establishing causality or temporal relationships between plasma NTN‐1 levels and PD symptoms. Longitudinal studies are needed to elucidate the dynamic changes in NTN‐1 concentrations throughout the disease course and their impact on symptom progression. Furthermore, potential confounding factors such as age, gender, medication use, and comorbidities should be carefully considered in future analyses to ensure the validity of the observed correlations.

In conclusion, the significant reduction in plasma NTN‐1 levels in PD patients compared to HC and their correlation with disease severity represent significant advancements in identifying reliable biomarkers and potential therapeutic targets for PD. This study contributes to understanding NTN‐1's involvement in neurodegenerative processes and highlights its potential as a disease marker. Future research should explore NTN‐1's precise role, therapeutic potential, and biomarker validity in larger, diverse cohorts. As our understanding of NTN‐1 in PD deepens, it may inform more personalized and effective disease management strategies.

## AUTHOR CONTRIBUTIONS

Ye Hua, Yi Fan, and Weifeng Lu performed study concepts and design. Ye Hua, Qingyu Yao, Bin Hu, and Feng Lu designed and performed clinical studies. Min Wang performed the ELISA experiments. Ye Hua and Yi Fan provided analysis and interpretation of data and statistical analysis. Ye Hua and Yi Fan performed writing, reviewing, and revising the paper. All authors read and approved the final paper.

## CONFLICT OF INTEREST STATEMENT

The authors declare that they have no conflict of interest.

## Data Availability

The raw data supporting the conclusions of this article will be made available by the authors, without undue reservation, to any qualified researcher.
